# Subject-specific pulse wave propagation modeling: Towards enhancement of cardiovascular assessment methods

**DOI:** 10.1371/journal.pone.0190972

**Published:** 2018-01-11

**Authors:** Jan Poleszczuk, Malgorzata Debowska, Wojciech Dabrowski, Alicja Wojcik-Zaluska, Wojciech Zaluska, Jacek Waniewski

**Affiliations:** 1 Department for Mathematical Modeling of Physiological Processes, Nalecz Institute of Biocybernetics and Biomedical Engineering, Polish Academy of Sciences, Warsaw, Poland; 2 Department of Anesthesiology and Intensive Therapy, Medical University of Lublin, Lublin, Poland; 3 Department of Physical Therapy and Rehabilitation, Medical University of Lublin, Lublin, Poland; 4 Department of Nephrology, Medical University of Lublin, Lublin, Poland; University of Colorado Denver School of Medicine, UNITED STATES

## Abstract

Cardiovascular diseases are the leading cause of death worldwide. Pulse wave analysis (PWA) technique, which reconstructs and analyses aortic pressure waveform based on non-invasive peripheral pressure recording, became an important bioassay for cardiovascular assessment in a general population. The aim of our study was to establish a pulse wave propagation modeling framework capable of matching clinical PWA data from healthy individuals on a per-subject basis. Radial pressure profiles from 20 healthy individuals (10 males, 10 females), with mean age of 42 ± 10 years, were recorded using applanation tonometry (SphygmoCor, AtCor Medical, Australia) and used to estimate subject-specific parameters of mathematical model of blood flow in the system of fifty-five arteries. The model was able to describe recorded pressure profiles with high accuracy (mean absolute percentage error of 1.87 ± 0.75%) when estimating only 6 parameters for each subject. Cardiac output (CO) and stroke volume (SV) have been correctly identified by the model as lower in females than males (CO of 3.57 ± 0.54 vs. 4.18 ± 0.72 L/min with p-value < 0.05; SV of 49.5 ± 10.1 vs. 64.2 ± 16.8 ml with p-value = 0.076). Moreover, the model identified age related changes in the heart function, i.e. that the cardiac output at rest is maintained with age (r = 0.23; p-value = 0.32) despite the decreasing heart rate (r = −0.49; p-value < 0.05), because of the increase in stroke volume (r = 0.46; p-value < 0.05). Central PWA indices derived from recorded waveforms strongly correlated with those obtained using corresponding model-predicted radial waves (*r* > 0.99 and *r* > 0.97 for systolic (SP) and diastolic (DP) pressures, respectively; *r* > 0.77 for augmentation index (AI); all *p*—*values* < 0.01). Model-predicted central waveforms, however, had higher SP than those reconstructed by PWA using recorded radial waves (5.6 ± 3.3 mmHg on average). From all estimated subject-specific parameters only the time to the peak of heart ejection profile correlated with clinically measured AI. Our study suggests that the proposed model may serve as a tool to computationally investigate virtual patient scenarios mimicking different cardiovascular abnormalities. Such a framework can augment our understanding and help with the interpretation of PWA results.

## Introduction

Over the last several decades pulse wave analysis (PWA) and pulse wave velocity (PWV) techniques became a well-recognized non-invasive tools to assess cardiovascular state, with PWV being the gold-standard for the measurement of arterial stiffness [1, 2]. The clinical benefits of using PWA to assess the cardiovascular risk and the impact of pharmacological intervention on the central blood pressure have been clearly shown in clinical trials [3, 4]. Those two techniques, however, are not interchangeable and they are limited by the scarce data of reference values for a healthy population [5]. There is only limited discussion about validity of the PWV technique, which typically relies on gating of pressure waveforms recorded using applanation tonometry with simultaneous electrocardiographs [5]. There are, however, concerns about the accuracy of PWA method, which attempts to reconstruct and then analyze the pressure waveform in the ascending aorta using non-invasive recordings of peripheral pressure profiles [6–10].

**Fig 1:**
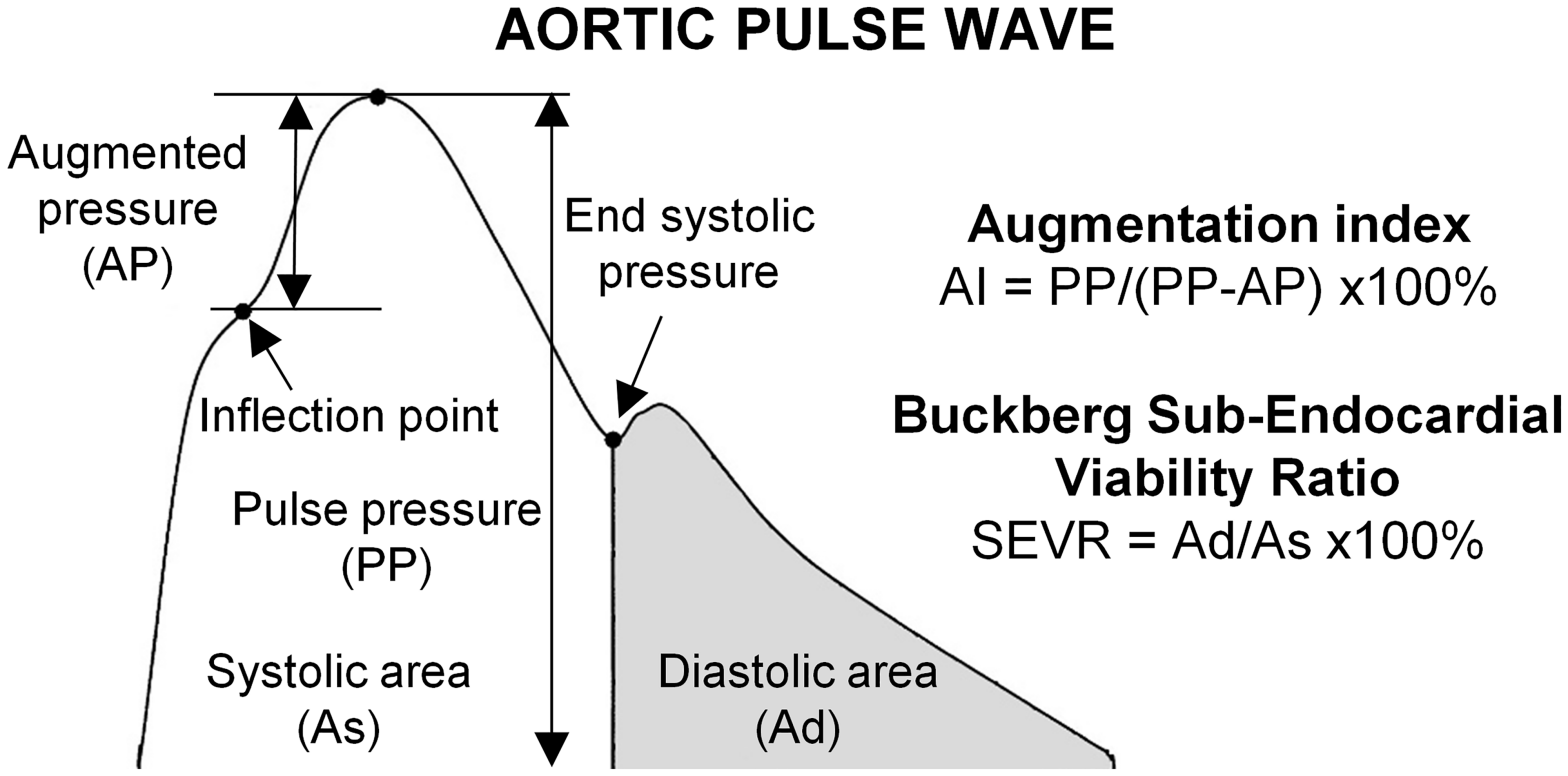
Features of the pulse wave in ascending aorta. Superposition of forward and reflected pressure waves results in certain amount of augmented pressure, with a characteristic inflection point located to the left of pressure peak in elderly subjects. In younger subjects inflection point is typically found to the right of pressure peak, what results in negative values of augmented pressure.

The PWA method relies on the usage of generalized transfer functions that attempt to describe the relation between peripheral and central pressure waveforms in the whole adult population [11]. However, since the appearance of the first study proposing this approach [12] and despite some additional validating studies [13–15] there is an ongoing debate about its validity as it is well recognized that there are some inter-patient and inter-groups differences in transfer function [6–10]. The quality of central pressure waveform reconstruction is especially important for the phase of the PWA during which some indices characterizing the wave, in addition to systolic and diastolic pressures, are being looked for [5]. One of such indices, which is associated with aging and cardiovascular risk [16–19], is augmentation index (AI) that represents the augmentation of central pressure height that is being introduced by the reflected waves [20], compare Fig 1.

Pulse wave analysis technique is especially important for individuals having high cardiovascular risk, such as patients with renal failure. However, various pathological conditions associated with chronic kidney disease and hemodialysis therapy, such as fluid overload, changes in vascular resistance, vasodilatory status, and the presence of surgically created arteriovenous blood access for hemodialysis may have a significant impact on the pulse-wave propagation through the arterial tree, and hence they could influence the PWA results. Dissection of information present in the shape and velocity of pulse wave would not be possible without a mathematical model. In the literature one can find many detailed physiology-based mathematical models of arterial blood flow that, if successfully calibrated with data, could help to understand better the abovementioned problems with PWA and ultimately lead to better and more patient-specific approaches. Existing models differ in complexity, starting from the simplest lumped models [21, 22], to more complicated distributed and 1D pulse wave propagation models [23–26], finishing with those simulating flow in full 3D setting [27, 28]; see [29] for the review and history of pulse wave propagation modeling. Limitations in computational power, however, restrict the application of 3D approaches only to a local segment of the arterial tree. There have been quite successful attempts to compare 1D and distributed pulse wave propagation models with clinical data [30–34]. However, to our knowledge, there have been no attempts to closely match predictions of such whole-tree models with PWA data collected in the clinic using commercially available device.

**Fig 2:**
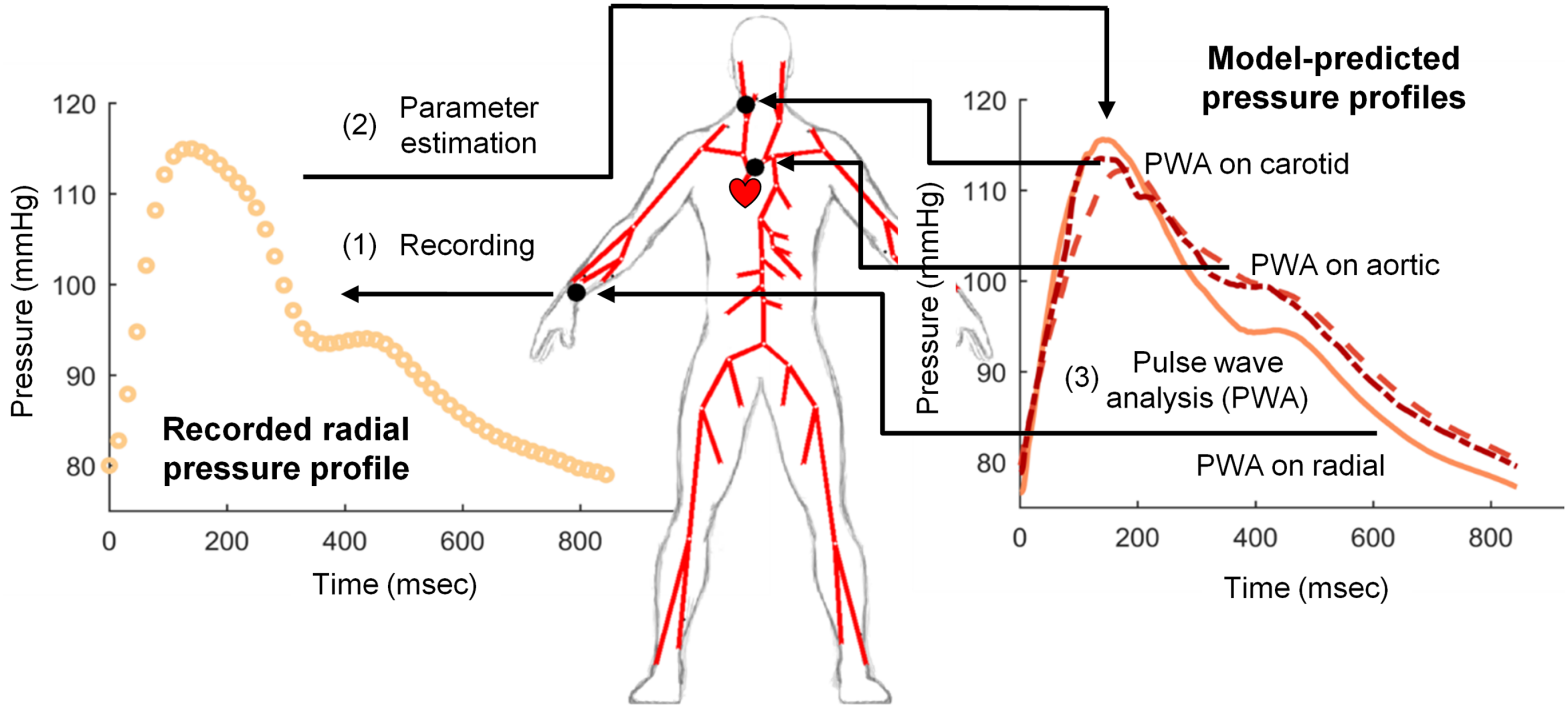
Schematic representation of the study workflow. (1) Radial pressure waveforms were recorded in 20 healthy subjects using applanation tonometer. (2) Recorded pressure waveforms were used to estimate parameters of the blood flow model in the system of 55 compliant arteries. (3) Pulse wave analysis using SphygmoCor software was performed on model-predicted radial, carotid, and aortic pressure waveforms separately.

In the paper we set out to investigate whether an 1D model of pulse wave propagation in the arterial tree can reproduce applanation tonometry recordings of the pressure waves in the radial artery and if corresponding model-predicted peripheral waves are providing results comparable to clinical readouts when used in pulse wave analysis, see Fig 2 for schematic workflow of the study. We analyze also the difference between central pressure waveforms reconstructed using generalized transfer function and those predicted by the whole arterial tree model. Our goal is to establish a computational framework that could be later used to theoretically investigate the validity of PWA method for groups having various cardiovascular system pathologies, such as patients with renal failure.

## Materials and methods

### Study subjects and clinical data

Radial pressure waveforms from 20 healthy individuals (10 males and 10 females) aged 27–61 years were recorded using applanation tonometry (SphygmoCor, AtCor Medical, Australia) with the participant comfortably seated. Volunteers were recruited between August 2015 and December 2016. The only enrollment criterion was lack of any diagnosed cardiovascular disease. The demographic and clinical characteristics of the study participants are shown in Table 1. All recordings were performed by one trained investigator at the Medical University of Lublin (Poland). The only criterion for measurement exclusion was insufficient quality of the recording defined and calculated by SphygmoCor software as Operator Index, but all of the recordings had the value of this index above recommended threshold of 74 (93 ± 5 range [82–100] for males; 87 ± 7 range [76–95] for females). Brachial blood pressure levels, which are required by SphygmoCor software to calibrate recorded peripheral waves, were measured with a Omron M3 Comfort automatic oscillometric upper arm blood pressure monitor (Omron Healthcare, Ltd., Kyoto, Japan). All of the participant gave an written consent and the study was approved by the Bioethical Committee at the Medical University of Lublin (Poland).

**Table 1:** Participant characteristics. Shown are means ± standard deviations and ranges.

Variables	Men (N = 10)	Women (N = 10)
Age, years	41.4 ± 9.95 [27 − 54]	42.2 ± 9.94 [31 − 61]
Height, cm	183.7 ± 8.68 [170 − 198]	164.6 ± 4.09 [158 − 172]***
Weight, kg	95.8 ± 17.93 [64 − 126]	62.6 ± 8.87 [49 − 75]***
Body Mass Index (BMI), kg/m2	28.21 ± 3.75 [22.15 − 32.77]	23.07 ± 2.91 [18.22 − 25.58]**
Brachial systolic pressure (SP), mmHg	133.7 ± 12.74 [112 − 154]	123.7 ± 10.85 [110 − 146]
Brachial diastolic pressure (DP), mmHg	83.4 ± 7.92 [72 − 96]	78.1 ± 5.51 [65 − 85]

** significantly different than in Men (p-value < 0.01),

*** significantly different than in Men (p-value < 0.001).

### Model of arterial tree geometry

We model the blood flow in a bifurcating binary tree of fifty-five larger systemic arteries in which individual vessels are axisymmetric elastic cylinders tapering along their length; see definition of the tree in Table 2. Geometry of the considered tree is based on the papers by Stergiopulos et al. [26] and Olufsen et al. [25] and allows to capture most important aspects of the arterial tree without substantially increasing the computational burden of the problem. Vessel’s tapering is modeled by imposing the equation describing its radius (*r*0 (*x*)) at the nominal pressure *P*_0_

$$\begin{array}{c} r_{0}\left(x\right)=r_{\text{in}}{\left(\frac{r_{\text{out}}}{r_{\text{in}}}\right)}^{x/L}\;, \end{array}$$ (1)

where *r*_in_ and *r*_out_ are the proximal and distal radii of the artery, respectively, and *L* is the length of the artery [25].

**Table 2:** Arterial tree structure definition based on papers by Stergiopulos et al. [26] and Olufsen et al. [25]. Parameters *r*_in_ and *r*_out_ are the proximal and distal radii of the artery, respectively. Resistance (*R*_*T*_ 10^4^g/s/cm^4^) and compliance (*C*_*T*_ 10^-6^g/s/cm^4^) are defined only for terminal arteries, see Eq (7). R and L denote right and left, respectively.

ID	Artery name	Length (cm)	*r*_in_ (cm)	*r*_out_ (cm)	Parent ID	*R*_*T*_	*C*_*T*_
1	Ascending aorta	4	1.525	1.42	-	-	-
2	Aortic arch	3	1.42	1.342	1	-	-
3	Brachiocephalic	4	0.95	0.7	1	-	-
4, 15	R+L Subclavian	4	0.425	0.407	3, 10	-	-
5, 11	R+L Com. carotid	17	0.525	0.4	3, 2	-	-
6, 16	R+L Vertebral	14	0.2	0.2	4, 15	4.79	1.32
7, 17	R+L Brachial	40	0.407	0.25	4, 15	-	-
8, 19	R+L Radial	22	0.175	0.175	7, 17	4.41	1.09
9, 18	R+L Ulnar	22	0.175	0.175	7, 17	-	-
10	Aortic arch	4	1.342	1.246	2	-	-
12	Thoracic aorta	6	1.246	1.124	10	-	-
13	Thoracic aorta	11	1.124	0.924	12	-	-
14	Intercostals	7	0.63	0.5	12	1.33	5.43
20	Celiac axis	2	0.35	0.3	13	-	-
21	Hepatic	2	0.3	0.25	20	-	-
22	Hepatic	7	0.275	0.25	20	3.86	2.38
23	Gastric	6	0.175	0.15	21	5	1.29
24	Splenic	6	0.2	0.2	21	2.15	5.47
25	Abdominal aorta	5	0.924	0.838	13	-	-
26	Superior mesenteric	5	0.4	0.35	25	0.92	23.91
27	Abdominal aorta	2	0.838	0.814	25	-	-
28, 30	R+L Renal	3	0.275	0.275	27, 29	1.03	13.92
29	Abdominal aorta	2	0.814	0.792	27	-	-
31	Abdominal aorta	13	0.792	0.627	29	-	-
32	Inferior mesenteric	4	0.2	0.175	31	6.84	0.85
33	Abdominal aorta	8	0.627	0.55	31	-	-
34, 47	R+L External iliac	6	0.4	0.37	33	-	-
35, 48	R+L Femoral	15	0.37	0.314	34, 47	-	-
36, 49	R+L Internal iliac	5	0.2	0.2	34, 47	6.4	0.75
37, 50	R+L Deep femoral	11	0.2	0.2	35, 48	4.65	1.49
38, 51	R+L Femoral	44	0.314	0.2	35, 48	-	-
39, 40, 52, 53	R+L Ext. + Int. carotid	16	0.275	0.2	5, 11	16.25	0.19
41, 54	R+L Post. tibial	32	0.125	0.125	38, 51	5.47	0.91
42, 55	R+L Ant. tibial	32	0.125	0.125	38, 51	4.51	1.23
43, 46	R+L Interosseous	7	0.1	0.1	9, 18	8.68	0.33
44, 45	R+L Ulnar	17	0.2	0.2	9, 18	6.32	1.08

### Constitutive model equations in each arterial segment

We model spatiotemporal changes in the cross-sectional area of the artery, *A*(*t*, *x*), and blood flow, *Q*(*t*, *x*), where *x* is the distance from the proximal end of the artery, under the assumption that each vessel is an impermeable axisymmetric elastic cylinder and blood is an incompressible fluid with given density, *ρ*, and viscosity, *μ*. We further assume, as in [23, 35], that the blood velocity profile is parabolic across artery cross-sectional area (Poiseuille profile) and, analogously to [25], we define the following relation between pressure exerted on artery wall (*P*, g/(s^2^cm)) and artery cross-sectional area

$$\begin{array}{c} P\left(t,x\right)-P_{0}=f\left(x\right)(1-\sqrt{\frac{A_{0}\left(x\right)}{A(t,x)}})\;, \end{array}$$ (2)

where function *f*(*x*) describes vessel’s wall elasticity and is expressed as

$$\begin{array}{c} f\left(x\right)=\frac{4}{3}(k_{1}\exp\left(k_{2}r_{0}\left(x\right)\right)+k_{3})\;. \end{array}$$ (3)

with parameters *k*_*i*_ being global, i.e. the same for each artery. Following standard derivation presented in detail in [25], we use the following continuity

$$\begin{array}{c} \frac{\partial A(t,x)}{\partial t}+\frac{\partial Q(t,x)}{\partial t}=0 \end{array}$$ (4)

and momentum

$$\begin{array}{c} \frac{\partial Q(t,x)}{\partial t}+\frac{\partial }{\partial x}(\frac{Q{\left(t,x\right)}^{2}}{A(t,x)})+\frac{A(t,x)}{\rho }\frac{\partial P(t,x)}{\partial x}=-\frac{8\pi \mu }{\rho }\frac{Q(t,x)}{A(t,x)} \end{array}$$ (5)

equations. Eqs (1)–(5) define the model and are used to obtain blood flow *Q* and pressure *P* in each arterial segment.

### Boundary conditions

In addition to Eqs (1)–(5) we need to impose aortic inflow, that is inflow condition at the inlet of ascending aorta (heart ejection profile), conditions at the tree bifurcations, and outflow conditions at the terminal ends of the arterial tree [29].

**Fig 3:**
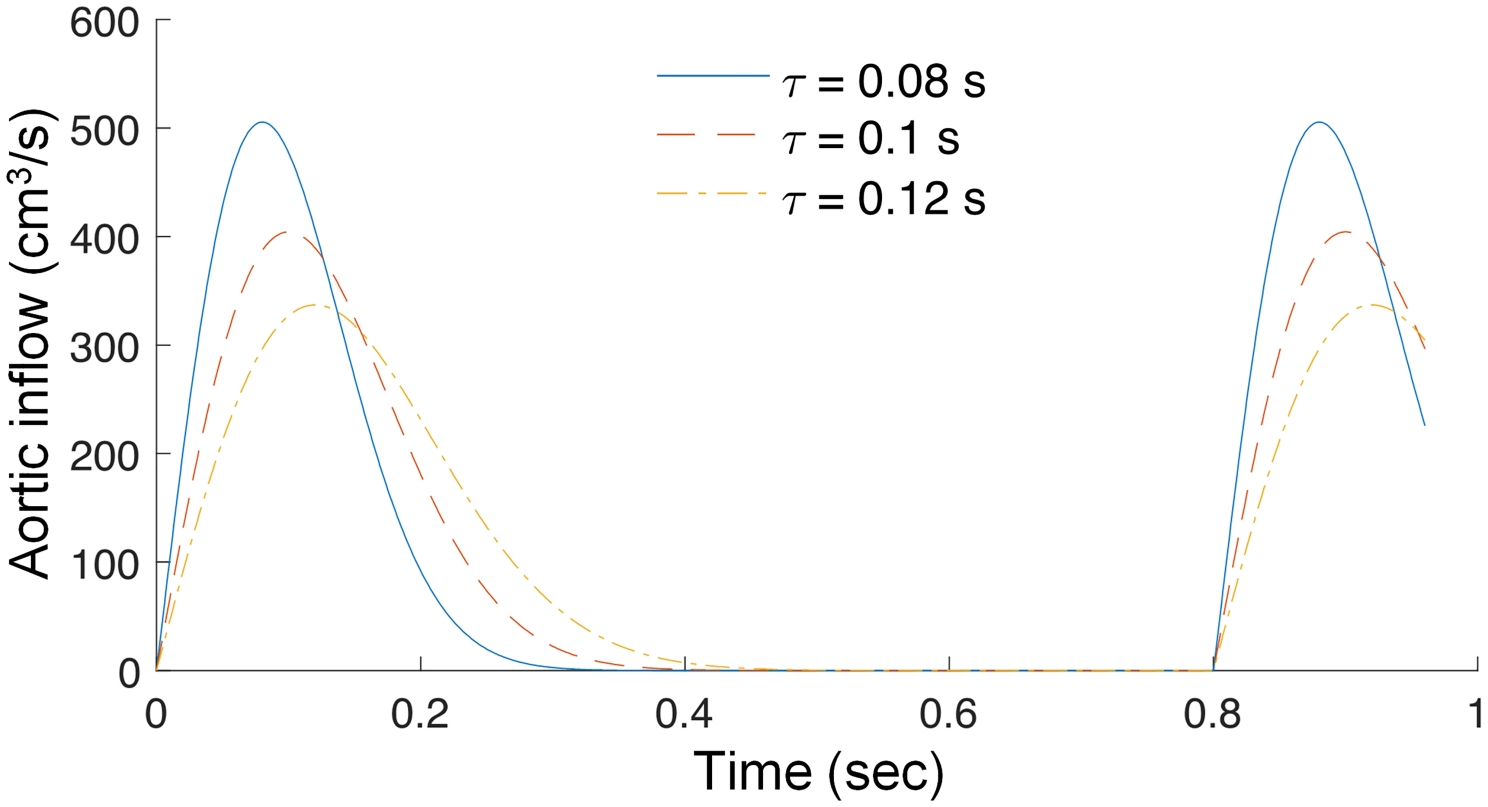
Assumed aortic inflow. Dependence of the heart ejection profile on parameter *τ* (*τ* = 0.08, 0.1 and 0.12 seconds) for 5 L/min cardiac output and heart rate of 75 beats/min, see Eq (6).

For the aortic inflow we need to define a time dependent flow caused by the cyclic heart contractions. In the literature one can find a number of different approaches, such as imposing ejection profile obtained from clinical measurement [25] or predicted by separate models of the heart [30, 33]. However, in order to reduce the complexity of the model and the number of subject-specific parameters we assume the following parametrized heart ejection profile

$$\begin{array}{c} Q_{in}\left(t\right)=\frac{\text{CO}}{\text{HR}\cdot \tau^{2}}\bar{t}\exp(-\frac{{\bar{t}}^{2}}{2\tau^{2}})\;,\;\;\bar{t}=t\;\mathrm{mod}\;T \end{array}$$ (6)

where CO is cardiac output per minute, HR is the heart rate, *T* = 1/*HR* is the length of cardiac period, and *τ* is the time at which ejection has its peak [36]; compare Fig 3.

At each end of terminal arterial segments we impose a three-element Windkessel model that can be expressed as the following differential relation between the terminal flow *Q*_*end*_ and the pressure *P*_*end*_

$$\begin{array}{c} R_{1}R_{2}C_{T}\frac{dQ_{end}\left(t\right)}{dt}=R_{2}C_{T}\frac{dP_{end}\left(t\right)}{dt}+(P_{end}\left(t\right)-P_{T})-(R_{1}+R_{2})Q_{end}\left(t\right)\;, \end{array}$$ (7)

where *R*_1_ + *R*_2_ = *R*_*T*_, *R*_1_/*R*_*T*_ = 0.2, *P*_*T*_ is the pressure at the venous end of the arterial tree, and *R*_*T*_ and *C*_*T*_ are total resistance and compliance of the terminal branches, respectively [26, 37]; see Table 2 for detailed values. At the bifurcation points we assume that there is no leakage, and hence the outflow from the parent vessel (*p*) must be balanced by the inflow into the daughter vessels (*d*_1_ and *d*_2_)

$$\begin{array}{c} Q_{end,p}=Q_{in,d_{1}}+Q_{in,d_{2}}\;, \end{array}$$ (8)

There is some loss of energy at the bifurcation points, which can be measured experimentally and expressed in the model in terms of loss coefficients [25]. It was shown, however, that assuming pressure continuity at the bifurcation point, i.e.

$$\begin{array}{c} P_{end,p}=P_{in,d_{1}}=P_{in,d_{2}}\;, \end{array}$$ (9)

is a good approximation in the considered setting [23, 25, 26]. Thus, we assume the above condition at each bifurcation point in the whole arterial tree.

### Parameter estimation procedure

The goal of the parameter estimation procedure was to find model parameters for which model-predicted radial pressure waveforms correspond the best to those recorded using applanation tonometry, i.e. for each subject separately we searched for parameters values that minimized the error

$$\begin{array}{c} \text{ERR}=\Delta {\text{SP}}^{2}+\Delta {\text{DP}}^{2}+\Delta P_{T1}^{2}+\Delta P_{T2}^{2}+\Delta P_{ED}^{2}+\Delta {\text{MP}}^{2}+\sum \Delta P{\left(t_{i}\right)}^{2}, \end{array}$$ (10)

where Δ denotes the difference between clinical measurement and model solution in radial artery (the latter scaled to the model-predicted pressure in brachial-cuff), SP is systolic pressure, DP is diastolic pressure, *P*_*T*1_ is the pressure at SphygmoCor estimated peak of the primary left-ventricular ejection pressure, *P*_*T*2_ is the pressure at the SphygmoCor estimated peak of the arterial reflection wave, *P*_*ED*_ is pressure at the SphygmoCor estimated end of ejection moment, MP is mean pressure, and *P*(*t*_*i*_) are pressure values at points sampled every 50 msec. Obviously, the above payoff functional is chosen arbitrarily, and one could consider minimizing the squared sum of differences at each recorded point of pressure wave. We decided to formulate hybrid payoff functional which, in addition to downsampled profile, takes into account the most important characteristic wave landmarks in order to avoid local minima in which only a part of the wave is fitted accurately. Each subject’s arterial tree was scaled compared to the nominal tree using parameter *S*, which is the ratio of subject height to the 175 cm height of the person with nominal arterial tree (see Table 2), i.e. length, proximal and distal radii of each vessel were multiplied by *S*. In addition, because the terminal resistance and compliance depends, among others, on the size of the tree that spans after the artery is truncated, we multiply terminal resistances and compliances by 1/*S*^3^ and *S*^3^, respectively [25]. The fitting procedure iteratively minimized the error (Eq (10)) by adjusting the values of the following main parameters: *k*_1_ and *k*_3_ describing global relation between the wall elasticity and the artery radius (Eq (3)), cardiac output (CO), and moment of the heart ejection peak *τ* (see Eq (6)). In addition to those parameters, fitting procedure was allowed to scale simultaneously all terminal resistances by factor *S*_*R*_ and compliances by the factor *S*_*C*_. For each subject, we started the optimization procedure with a population of 24 sets of 6 parameters each, which were then iteratively modified towards a better solution using heuristic Particle Swarm Optimization method [38] for 100 iterations. After those initial iterations each resulting parameter set served as an initial point for a gradient-based trust-region-reflective algorithm, which, in order to minimize the error, uses a quadratic approximation for the minimized function in a small neighborhood (trust region) around the current point [39]. Connecting the heuristic and deterministic procedure typically allows to speed up the convergence and helps to avoid local minima.

### Pulse wave analysis

SphygmoCor software allows to perform PWA only on the radial, aortic and carotid waveforms, with the restriction that the last one has not been approved by FDA (message displayed by the software). Thus, in order to perform pulse wave analysis on the model-predicted radial, carotid and aortic pressure waveforms we used SphygmoCor software in the simulation mode, i.e. 20 cycles of model solution were written to a proprietary file format that was later treated by SphygmoCor software as an input signal. In the case of aortic pressure waveform SphygmoCor software does not process the wave and characteristic indices are calculated directly on the input. In Fig 1 we present schematic representation of PWA reconstructed aortic waveform together with the formulas for reported indices.

### Statistical methods

The data are presented as mean ± SD unless stated otherwise. Univariate statistical dependence between variables was measured by Pearson’s correlation coefficient (r). To investigate statistical differences we used two-sided paired Wilcoxon signed rank test with continuity correction. Statistical significance was set at the level of p-value = 0.05 unless otherwise indicated. Stepwise linear regression method with at most pairwise interactions model and R-squared based criterion for predictor addition or removal (0.1 threshold for adding and 0.05 for removing the term) was used to check whether there is any correlation between clinically measured AI and various combinations of model parameters.

## Results

### Data fitting and parameter estimation

**Fig 4:**
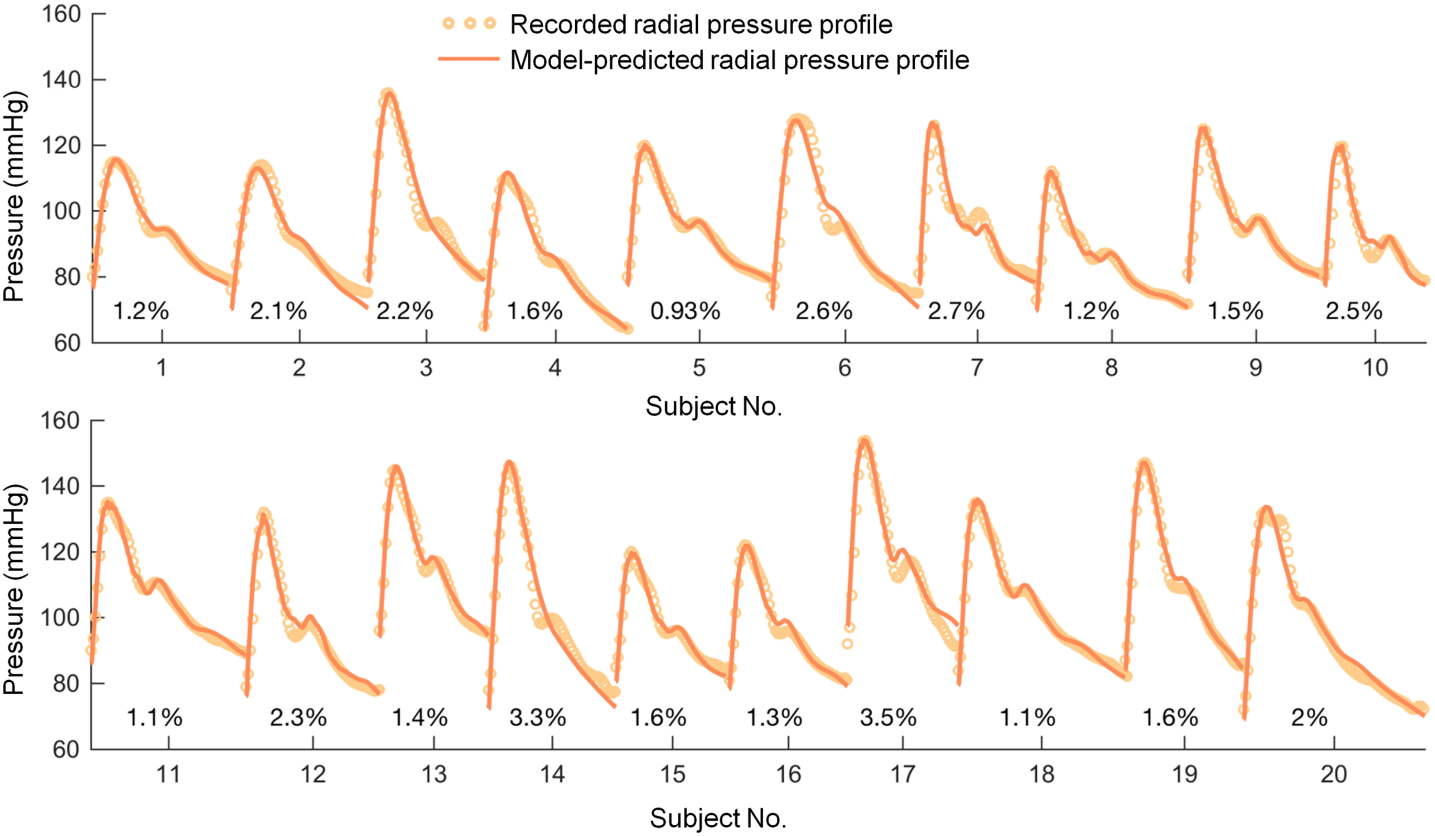
Comparison of pressure waveforms in radial artery recorded using SphygmoCor (circles) with the fitted model curves (solid lines) for 20 healthy volunteers. Shown are also mean absolute percentage errors for each subject separately. Summary of parameters used in the simulations is presented in Table 3.

The model was able to reproduce clinically measured radial waveform in each of the studied subjects (mean absolute percentage error of 1.87 ± 0.75%; compare Fig 4). Coefficient of determination was lower than 0.95 only in 3 subjects, with the lowest value of 0.89, indicating that the model is able to precisely account for most of the variance in measured pulse waveform. Moreover, none of the standard radial waveform characteristics, i.e. systolic, diastolic and mean pressures, were statistically different between those estimated by the model and those from recorded data, compare curves presented in Fig 4. The fixed and model-estimated parameters, with comparison between males and females, are listed in Table 3. In addition to difference in cardiac output, females had lower model-estimated stroke volume (SV) than males (SV = CO/HR; 49.5 ± 10.1 vs. 64.2 ± 16.8 ml) at the borderline significance level (p-value = 0.076).

**Table 3:** Fixed and estimated model parameters. For the parameters that were estimated means ± standard deviations are shown for Men and Women separately.

Parameter	Unit	Men (N = 10)	Women (N = 10)	Reference
Pressure at the venous end of the arterial tree (Eq (7)), *P*_*T*_	mmHg	15		Assumed
Blood density (Eq (5)), *ρ*	g/cm^3^	1.04		[37]
Blood viscosity (Eq (5)), *μ*	g/(cm s)	0.04		[37]
Reference distending pressure (Eq (2)), *P*_0_	mmHg	97		[26]
Heart rate (Eq (6)), HR	beats/min	67.8 ± 14.82	73 ± 6.57	from data
Vessel wall elasticity, (Eq (3))				
*k*_1_	10^7^g/(s^2^ cm)	0.97 ± 1.24	0.38 ± 0.28	Estimated (nominal value = 2)
*k*_2_	-	-22.53		[25]
*k*_3_	10^5^ g/(s^2^ cm)	8.88 ± 2.06	10.1 ± 6.07	Estimated (nominal value = 8.65)
Cardiac output (Eq (6)), CO	L/min	4.18 ± 0.72	3.57 ± 0.54*	Estimated (nominal value = 4.5)
Peak ejection moment (Eq (6)), *τ*	msec	90.4 ± 13.52	99.39 ± 14.15	Estimated (nominal value = 100)
Scaling of terminal resistances, *S*_*R*_	-	1.56 ± 0.39	1.17 ± 0.27*	Estimated (nominal value = 1)
Scaling of terminal compliances, *S*_*C*_	-	7.54 ± 2.07	10.35 ± 0.59*	Estimated (nominal value = 1)

*significantly different than in Men (p-value < 0.05)

We found number of significant correlations between estimated subject-specific model parameters and demographic/clinical characteristics of the participants, compare Table 4. The two parameters that correlate with all of the basic participant characteristics, except for age, are scaling factors for terminal resistances and compliances (*S*_*R*_ and *S*_*C*_, respectively). In addition, we found that age correlates only with parameters describing the heart ejection profile (heart rate, stroke volume, time to the peak of ejection) and systolic pressure in brachial artery depends on both the stiffness of the bigger arteries (parameter *k*_3_) and cardiac output. Investigation of pairwise correlations between estimated subject-specific model parameters revealed only negative correlation between heart rate and time to the peak of ejection profile (HR and *τ*, respectively; see Eq (6); *r* = −0.53 with p-value = 0.016).

**Table 4:** Pearson correlation coefficients between model-estimated parameters (see Table 3) and demographic/clinical participants characteristics (see Table 1).

	k1	k3	CO	*τ*	*S*_*R*_	*S*_*C*_	HR	SV
SP	0.096	0.57**	0.45*	-0.0017	0.57**	-0.56*	-0.061	0.35
DP	0.024	0.17	0.16	-0.33	0.67**	-0.62**	0.2	-0.041
Age	-0.15	0.25	0.23	0.61**	-0.071	0.10	-0.49*	0.46*
Height	0.35	0.014	0.50*	-0.22	0.72**	-0.71**	-0.30	0.55*
Weight	0.25	0.18	0.37	-0.15	0.8**	-0.69**	-0.17	0.40
BMI	0.15	0.33	0.20	-0.07	0.70**	-0.55*	-0.034	0.22

SP—Brachial systolic pressure, DP—Brachial diastolic pressure, BMI—Body Mass Index, SV—stroke volume (CO/HR);

*p-value < 0.05,

**p-value < 0.01

### Pulse wave analysis on recorded and model-predicted waveforms

**Fig 5:**
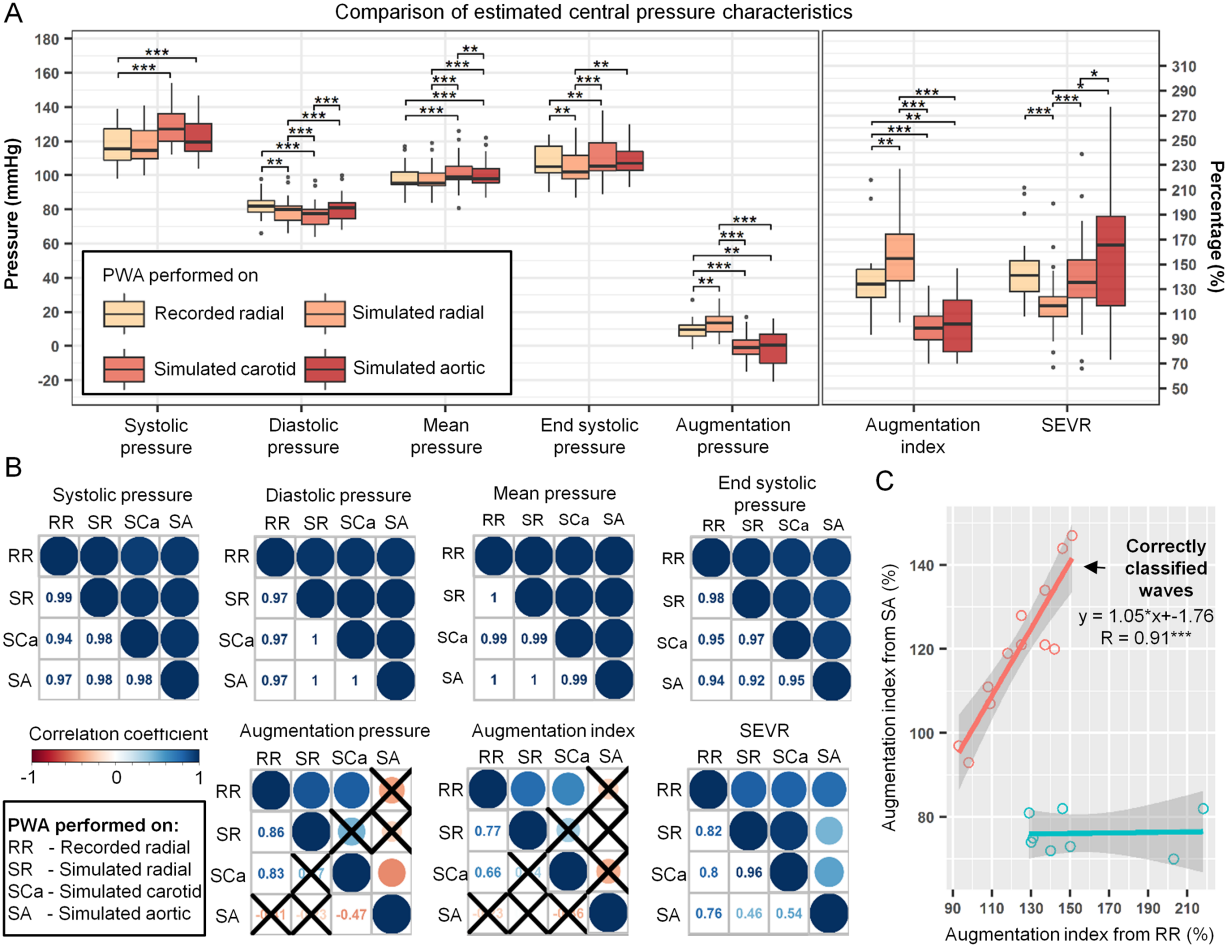
Comparison of the central pulse wave characteristics estimated using SphygmoCor software using different input pressure waveforms, i.e. recorded radial (RR), simulated radial (SR), simulated carotid (SCa), and simulated aortic (SA) waveforms. (A) Boxplots comparing pressure indices together with augmentation index and sub-endocardial viability ratio (SEVR). Symbols *, **, and *** denote p-value < 0.05, < 0.01 and < 0.001, respectively. (B) Pearson correlation coefficient matrices between different central waveform characteristics. Shown is a graphical representation of the correlation coefficient where size and color of the circle indicates correlation strength and direction (upper diagonal) together with correlation coefficients values (lower diagonal). Correlation coefficients with p-value ≥ 0.05 are crossed out. (C) Scatter plot and linear regression models comparing augmentation index estimated using recorded radial and simulated aortic pressure waveforms. Shown is also regression equation and Pearson correlation coefficient for the subgroup of patients for which both aortic waves were classified as the same type.

Comparison of PWA results performed on the recorded and simulated radial pressure profiles indicate that reconstructed central pressure indices are very sensitive to small differences in supplied peripheral pressure waveform shape, as statically significant differences were found for all of the investigated indices, except for systolic and mean pressures, compare Fig 5A. However, their values are similar and are highly interchangeable (r ≥ 0.77, p-value < 0.01; see Fig 5B).

Pulse wave analysis performed using SphygmoCor software on the simulated carotid and simulated central (aortic) pressure waves showed that the basic reconstructed central pressure indices, i.e. systolic, diastolic, mean and end diastolic pressures, are also similar to those obtained from recorded radial wave and highly interchangeable (r ≥ 0.94, p-value < 0.001). However, other central PWA-derived indices that depend highly on the characteristic wave landmarks locations, such as augmentation pressure and augmentation index, are significantly different between those reconstructed from recorded radial wave and those obtained from analyzing both simulated aortic and carotid waveforms, see Fig 5A. In case of augmentation pressure associated indices calculated using simulated carotid and aortic pressure waveforms, this difference can be mainly attributed to the inability of SphygmoCor software to locate the characteristic inflection point (see Fig 1) before the systolic peak in some of the subjects (see Fig 5C). This results in negative augmentation pressure, augmentation index below 100%, and apparent negative correlations. If one performs correlation analysis only in the cases in which inflection point was located on the same side of systolic peak as in case of PWA performed on recorded radial profile, then interchangeability is significant (see Fig 5C). Out of the 8 cases presented in Fig 5C that were classified incorrectly, 4 subjects had elevated pressure (brachial systolic pressure > 140 mmHg) and 1 subject had lower quality estimate for inflection point location reported by SphygmoCor when applied to recorded peripheral profile.

### Augmentation index is correlated to the heart ejection profile

**Fig 6:**
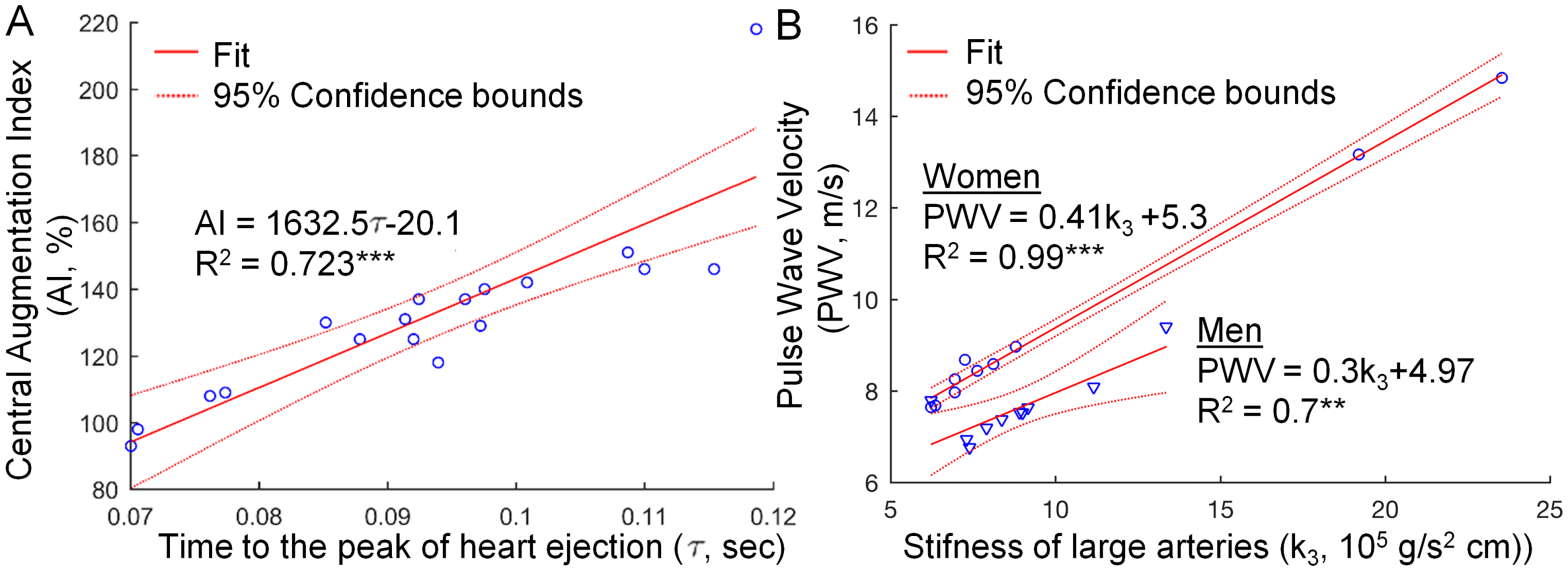
Dependence of most clinically relevant pulse wave indices on model parameters. (A)Resulting stepwise regression model for predicting central augmentation index. The steeper is the heart ejection profile, i.e. the smaller is the parameter *τ*, the smaller is the central augmentation index (AI) estimated using pulse wave analysis method. (B) Correlation between the model-predicted pulse wave velocity (PWV) and the model-estimated stiffness of large arteries described by parameter *k*_3_ (compare Eqs (2) and (3)). PWV was calculated by dividing the distance between aortic arch and femoral artery by the time needed by the wave to travel that path.

We performed stepwise regression starting from the linear model consisting of parameters *k*_1_, *k*_3_, 1/*k*_1_, 1/*k*_3_, *S*_*R*_, *S*_*C*_, CO, *τ*, and stroke volume as a predictors for central augmentation index derived using recorded radial pressure waveform. Interestingly, the procedure removed all predictors except for parameter *τ* which describes heart ejection profile (see Fig 6A), resulting in regression equation *AI* = 1632.5*τ*−20.107 (R^2^ = 0.739, Adjusted R^2^ = 0.723, p-value < 0.001).

## Discussion

The predictions of a physiology-based mathematical model of pulse wave propagation in the arterial tree were compared to a pulse wave analysis results performed using commercially available device on a group of 20 healthy volunteers. The first and the most crucial part of the study focused on fitting the model-predicted radial pressure waveforms to those recorded non-invasively using applanation tonometry. The optimization procedure looked for the values of six patient-specific parameters by taking into account characteristic landmarks of the peripheral waveform together with its overall shape. Subject-calibrated model successfully reproduced all measured radial profiles conserving all of the basic pressure indices such as diastolic, systolic and mean pressures. Interestingly, despite the lower average model-predicted cardiac output than the one reported in the literature [40], because not all of the arterial tree segments have been included in the model, the value of cardiac output has been correctly identified by the model as lower in females than males (3.57 ± 0.54 vs. 4.18 ± 0.72 L/min; p-value < 0.05). Compared to the nominal values of elasticity related parameters considered in [25], i.e. *k*_1_ = 2 × 10^7^ and *k*_3_ = 8.65 × 10^5^, our parameter estimation procedure predicts that bigger arteries have similar stiffness (estimated *k*_3_ is not statistically different than the nominal value, p-value 0.49) and stiffening effect with the decrease of vessel diameter is not as big as previously estimated (estimated *k*_1_ is about half and 1/5 of the nominal value for males and females, respectively; p-value < 0.001); compare Table 3. The model predicts also that the terminal resistances and terminal compliances are larger than previously assumed (p-value < 0.001).

Pairwise correlation analysis performed between subject-specific model parameters and the demographic/clinical characteristics, identified that systolic brachial pressure positively correlates with the stiffness of large arteries (parameter *k*_3_) and the cardiac output (Table 4.). However, the model identified that only resistances and compliances for the outflow from terminal branches of the arterial tree correlate with both systolic and diastolic brachial pressures. Interestingly, the model correctly predicted that there is a positive correlation between height and both cardiac output and stroke volume, Table 4, compare [41]. Moreover, the model properly identified age related changes in the heart function, i.e. that, because of the increase in the stroke volume, cardiac output at rest is maintained with age, despite the decreasing heart rate [42]; compare third row in Table 4. We expected also to find a correlation between the elasticity of the arterial walls and age, i.e. that the arteries are stiffening with age, but the p-value for that correlation was insignificant. This negative result, however, might be a consequence of small study group and more data might be required for validation.

Calibration of the model should result in pulse wave velocity (PWV) values within the clinically observed ranges. Thus, to additionally validate proposed framework we have calculated the pulse wave velocity for each patient by dividing the distance between aortic arch and femoral artery by the time needed by the wave to travel that path. As to be expected, we found that PWV closely correlates with stiffness of larger arteries (see Fig 6B). Moreover, its model-predicted average value of 8.5 ± 2.0 m/s is similar to 8.9 ± 1.8 m/s in males and 8.1 ± 2.0 m/s in females reported in [43] where PWV was examined by measuring the time difference of systolic pulse waves in arteries from the aortic arch to the popliteal artery using whole-body impedance cardiography.

Pulse wave analysis performed using SphygmoCor software on both recorded and simulated pressure profiles provided highly interchangeable results when considering basic central pressure waveform characteristic. However, correlations between indices involving calculation of characteristic waveform landmarks were significantly lower, indicating that PWA method is highly sensitive to small differences in supplied pressure profiles. This indicates that the model might need further extension by, for example, incorporating heart model as the inlet boundary condition [30, 33], considering more detailed structure of arterial tree [33, 37], or more detailed outflow conditions at the terminal ends [25].

We found that the systolic pressure of the model simulated aortic wave was on average higher by 5 mmHg than the one reconstructed by SphygmoCor software from radial profiles. The problem of central systolic pressure underestimation when using PWA with generalized transfer function approach has been previously reported in the literature [44, 45] (underestimation by 10–13 mmHg on average). This underestimation is typically attributed to the inaccuracies of the oscillometric brachial cuff measurements used in PWA for radial pulse waveform calibration, but our study suggests that at least some of this underestimation can be attributed to the inaccuracy of generalized transfer function itself. Recent studies have shown that PWA with its augmentation index is not a surrogate measure for pulse wave velocity and thus, it may not be the appropriate way to assess the arterial stiffness [17, 46, 47]. This observation is confirmed by our study as none of the subject-specific parameters related to the vessel wall elasticity (*k*_1_ and *k*_3_) correlated with the measured augmentation index. Interestingly, most of the variance in the PWA derived augmentation index is explained by the parameter describing the shape of the heart ejection profile (parameter *τ*), i.e. the steeper is the ejection profile the smaller is the augmentation index, Fig 6A. Slower ejection could indicate that the heart is working under higher workload and this could explain the correlation of augmentation index with cardiovascular risk factors [48].

Our results indicate that the model can serve as a framework to computationally investigate various virtual patient scenarios for pulse wave analysis methods. This is especially important for the cohorts with many additional cardiovascular pathologies such as patients with end stage renal disease, in which standard PWA might be affected by existence of arteriovenous (AV) fistula or substantial fluid overload. The creation of an AV fistula induces a substantial disturbance of systemic blood flow (fistula flow may be over 600 mL/min) and causes an adverse imbalance between subendocardial oxygen supply and increased oxygen demand following increased cardiac output [49]. In such cases the framework could help with derivation of more personalized transfer functions to increase PWA accuracy. Moreover, one could study the dependence of PWA results on the existence of local vasculopathy lesions by modifying geometry (Eq (1)) and elasticity (Eq (3)) for selected elements of the arterial tree. Finally, PWA method based on locating inflection points has recognized limitations [50] and predicted landmarks necessary to calculate AI do not correspond with those calculated using gold-standard Westerhof’s wave separation method [51]. The latter method requires information about blood flow in the aorta and thus the model, if validated prospectively, could provide a surrogate measure for central blood flow profile which would allow for utilization of more accurate wave separation analysis. Additional prospective validation of the model could be based on data from patients with left heart catheter or, if only non-invasive measurements are possible, using data from e.g. impedance cardiography and Doppler ultrasound test.
